# Synthesis and structure of 4,5-diphenyl-1*H*-imidazol-3-ium thio­cyanate

**DOI:** 10.1107/S2056989026005931

**Published:** 2026-06-09

**Authors:** Mohamed Abdellatif Bensegueni, Aouatef Cherouana

**Affiliations:** a Unité de Recherche de Chimie de l'Environnement et Moléculaire Structurale Université Constantine 1 Frères Mentouri Algeria; University of Aberdeen, United Kingdom

**Keywords:** crystal structure, 1*H*-imidazol-3-ium derivative, hydrogen bonds, Hirshfeld surface analysis

## Abstract

In the title mol­ecular salt, C_15_H_13_N_2_^+^·SCN^−^, the dihedral angles between the imidazole ring and pendant phenyl groups are 51.61 (14) and 35.47 (15)°. In the crystal, the ions are linked by N—H⋯N and C—H⋯S hydrogen bonds, forming *R*^4^_2_(16) loops. These units are further connected by C—H⋯π and C—S⋯π inter­actions into a three-dimensional supra­molecular network. Hirshfeld surface analysis of the imidazolium cation shows that H⋯H (42.9%), C⋯H/H⋯C (34.9%), N⋯H/H⋯N (10.7%) and H⋯S/S⋯H (6.5%) contacts are the major contributors to the crystal packing.

## Abstract 2

In the title salt, the components are linked by N—H⋯N, C—H⋯S, C—H⋯π and C—S⋯π inter­actions.

## 1. Chemical context

Imidazole derivatives continue to attract attention in structural and supra­molecular chemistry because they combine aromatic character, amphoteric behaviour and multiple donor/acceptor sites within a compact heterocyclic framework (Chen, 2016 ▸). In their neutral form, imidazoles are widely encountered as ligands and functional organic building blocks, whereas protonated imidazolium species are particularly attractive as cationic components of mol­ecular salts, where they can serve as efficient hydrogen-bond donors and promote the formation of extended supra­molecular assemblies. Aryl substitution at the 4- and 5-positions further enlarges the π-surface of the heterocycle and can enhance weak inter­molecular contacts involving aromatic rings.

A search of the Cambridge Structural Database (CSD, version 6.01 with updates to February 2026; Groom *et al.*, 2016 ▸) for the 4,5-di­aryl­imidazole skeleton shows that this family is structurally diverse and includes neutral mol­ecules, solvates and ionic derivatives. Representative neutral examples include 4,5-diphenyl-1*H*-imidazole (Stibrany *et al.*, 2004 ▸; Kounavi *et al.*, 2012 ▸), 2-(4,5-diphenyl-1*H*-imidazol-2-yl)phenol (Fridman *et al.*, 2009 ▸), 4-(4,5-diphenyl-1*H*-imidazol-2-yl)benzaldehyde (Kimura *et al.*, 2002 ▸), 2,4,5-triphenyl-4,5-di­hydro-1*H*-imidazole hemihydrate (Huang *et al.*, 2006 ▸).

Related salts and co-crystals further illustrate the versatility of this heterocyclic platform in the solid state. Examples include 4,5-diphenyl-2-(4-nitro­phen­yl)-1*H*-imidazole clathrates with water, acetic acid and dimethyl sulfoxide (Kaftory *et al.*, 1998 ▸), 2-(3-nitro­phen­yl)-4,5-diphenyl-1*H*-imidazol-3-ium nitrate (Zhang, 2009 ▸), and trans-2-amino-4,5-diphenyl-4,5-di­hydro­imidazolium nitrate derivatives (Wüstenberg *et al.*, 2023 ▸). These examples show that protonation of the imidazole ring and association with counter-ions or neutral conformers can generate a rich variety of supra­molecular arrangements governed by classical hydrogen bonds and weaker aromatic contacts.
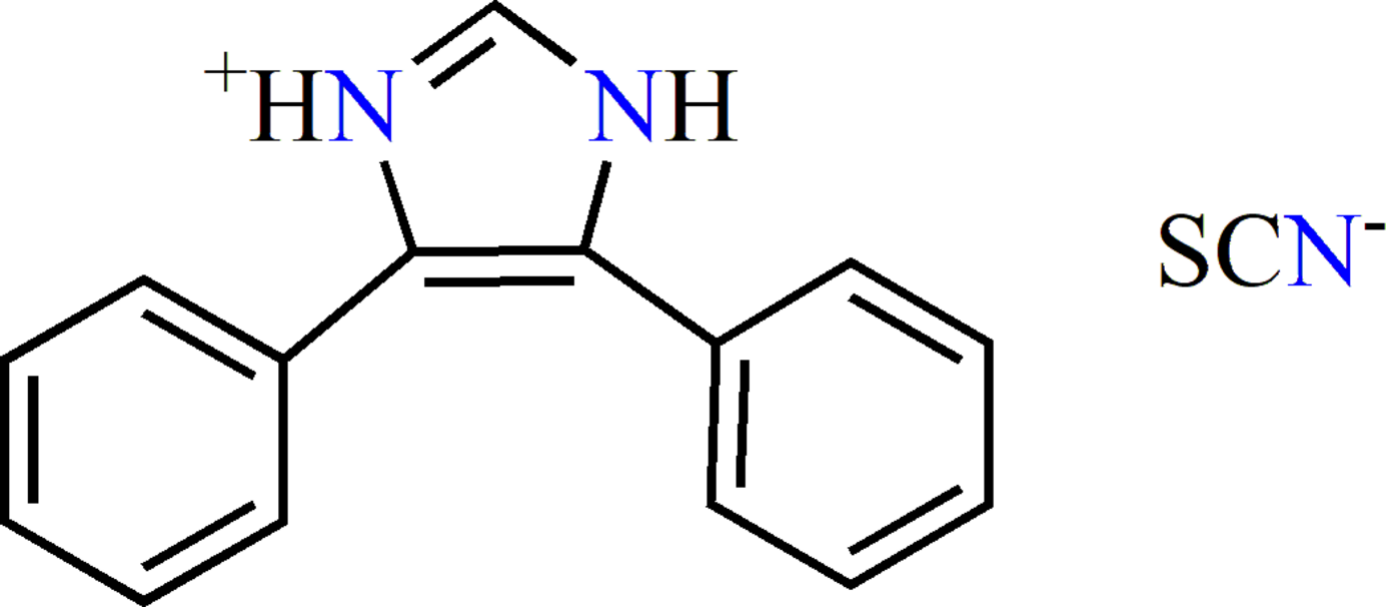


As part of our studies in this area, and in the context of our ongoing research on azole-based compounds (Bensegueni *et al.*, 2009 ▸, 2014 ▸, 2015 ▸, 2020 ▸), we now report the synthesis and structure of the title mol­ecular salt, C_15_H_13_N_2_^+^·SCN^−^ (**I**), in order to analyse how different types of hydrogen bonds cooperate in the consolidation of the crystal packing.

## 2. Structural commentary

The asymmetric unit of compound (**I**) consists of one 4,5-bi­phenyl­imidazolium cation and one thio­cyanate anion. The crystal structure is ortho­rhom­bic and crystallizes in the space group *P*2_1_2_1_2_1_ with a well-defined absolute structure.

**Figure 1:**
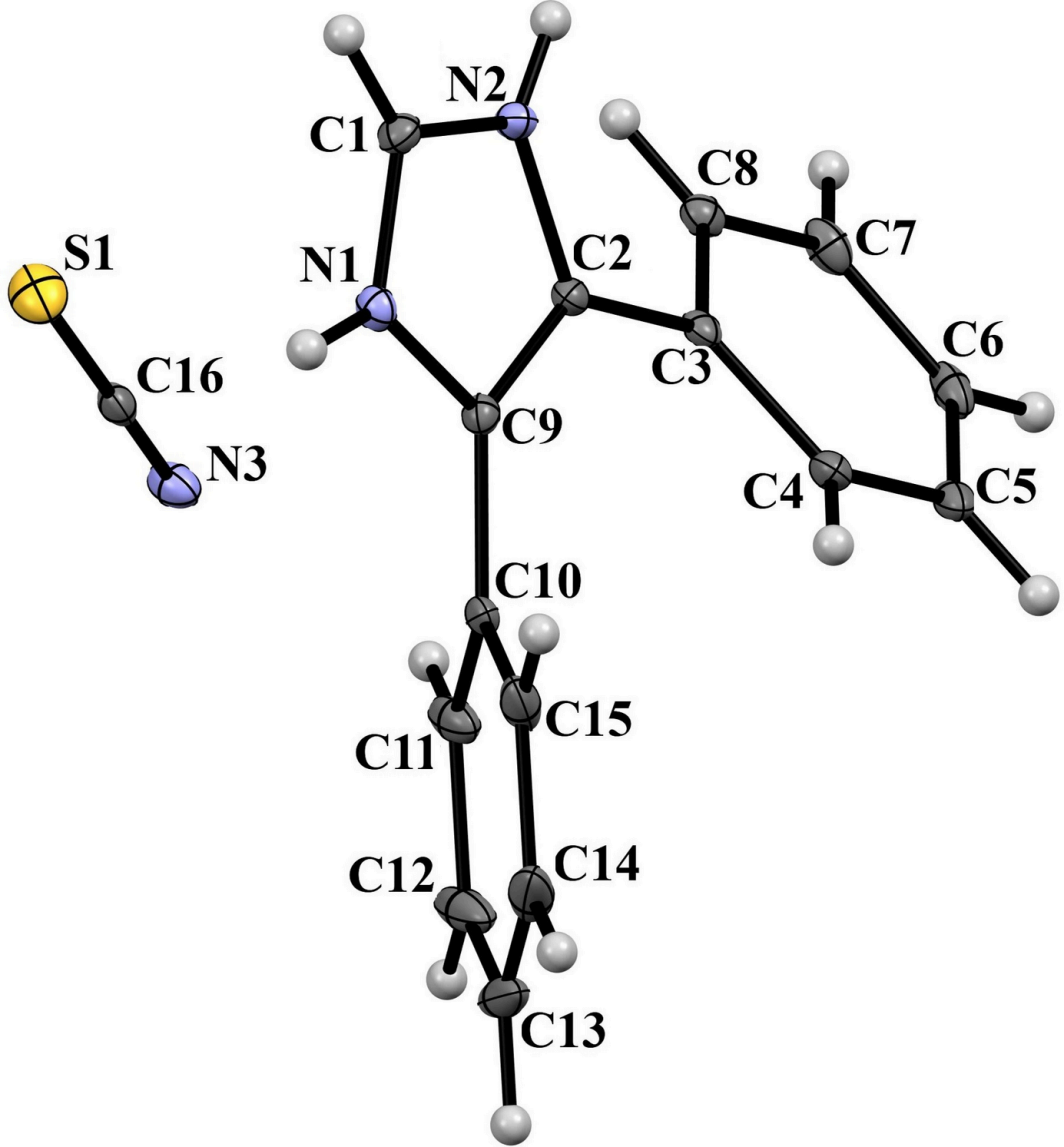
The mol­ecular structure of (**I**) showing displacement ellipsoids drawn at the 50% probability level.

The cation is formed by a protonated imidazolium ring substituted at the 4- and 5-positions by two phenyl groups. The imidazolium core, defined by atoms N1/N2/C1/C2/C9, displays bond lengths of 1.319 (4) Å for N1—C1, 1.326 (4) Å for N2—C1, 1.382 (4) Å for N1—C9 and 1.386 (3) Å for N2—C2, which are consistent with charge delocalization within the aromatic imidazolium fragment (Fig. 1 ▸). The cation consists of two phenyl rings (*A* containing C3–C8 and *C* containing C10–C15) and one imidazole ring (*B*) (see supplementary figure). The inter­planar angles are 51.61 (14)° for *A*/*B*, 35.47 (15)° for *B*/*C* and 54.80 (12)° for *A*/*C*.

As expected, the thio­cyanate anion is essentially linear, with an N3—C16—S1 bond angle of 179.4 (3)°. The corresponding bond distances, N3—C16 = 1.171 (4) Å and C16—S1 = 1.622 (3) Å, are in agreement with the expected geometry of a thio­cyanate anion.

## 3. Supra­molecular features

**Figure 2:**
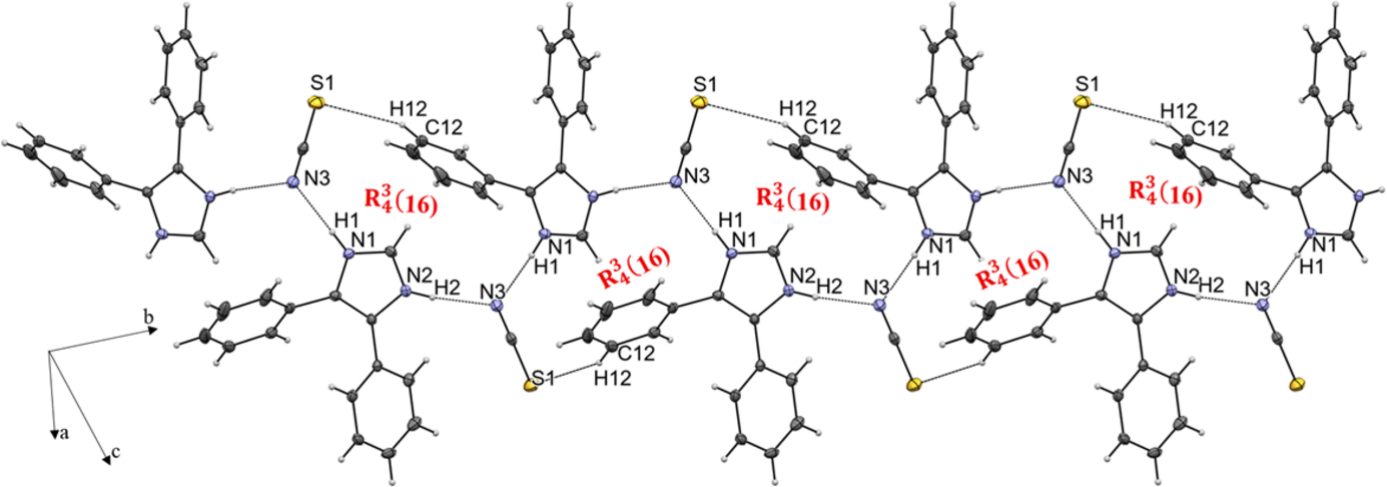
The crystal packing of (**I**), showing the 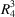
(16) hydrogen-bonded ring motifs and the one-dimensional chain generated by N—H⋯N and C—H⋯S inter­actions.

The crystal structure of (**I**) features three hydrogen bonds involving the thio­cyanate anion as the principal acceptor. The N1—H1*N*⋯N3 and N2—H2⋯N3 hydrogen bonds (Table 1 ▸), with H⋯N distances of 1.98 and 2.02 Å, together with the C12—H12⋯S1 contact (H⋯S = 2.93 Å), generate an *R*_4_^3^(16) ring motif that links two cations and two anions into discrete supra­molecular cycles in the crystal packing (Fig. 2 ▸). In addition to these classical hydrogen bonds, the structure features three C—H⋯π inter­actions, with each aromatic ring accepting one such inter­action. The C1—H1*A*⋯*Cg*3 contact, with a notably short H⋯*Cg* distance of 2.45 Å, reinforces the 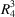
(16) motif (Etter *et al.*, 1990 ▸). The other two bonds connect adjacent motifs into extended chains (supplementary figure).

**Figure 3:**
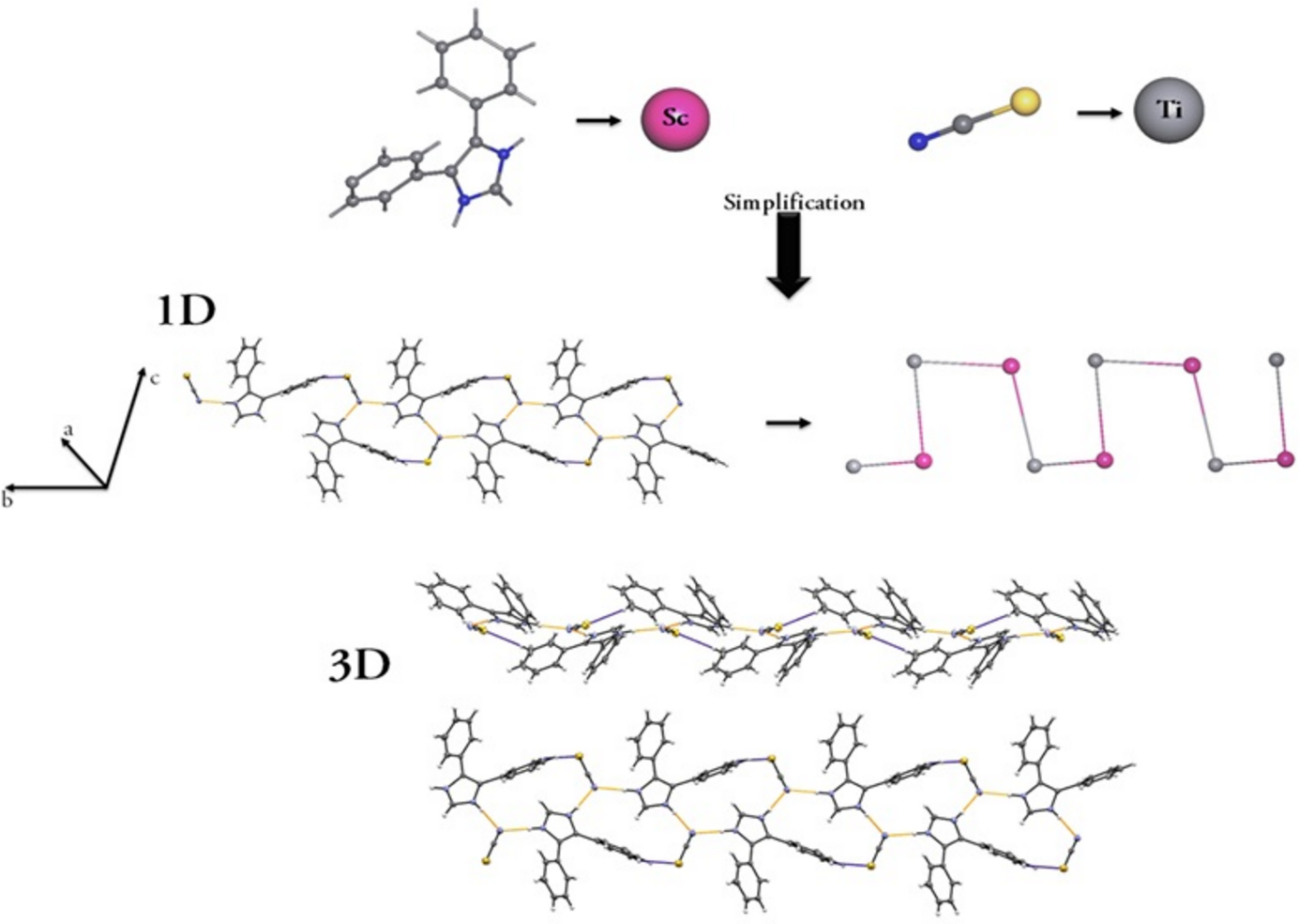
Simplified representation of the crystal packing showing the one-dimensional zigzag chain and its three-dimensional extension in (**I**).

The crystal packing also features a very weak π–π stacking inter­action between the *A* and *B* aromatic rings, with a centroid–centroid separation of 4.201 (12) Å, reinforced by a C—S⋯*Cg*1 contact [3.534 (2) Å, 94.96 (11)°], which links the hydrogen-bonded chains along the *a*- and *c*-axis directions and contributes to the three-dimensional supra­molecular architecture. Overall, these inter­actions produce a zigzag one-dimensional arrangement extending along the *b* axis, which is then assembled into the full three-dimensional network (Fig. 3 ▸).

## 4. Hirshfeld surface analysis

**Figure 4:**
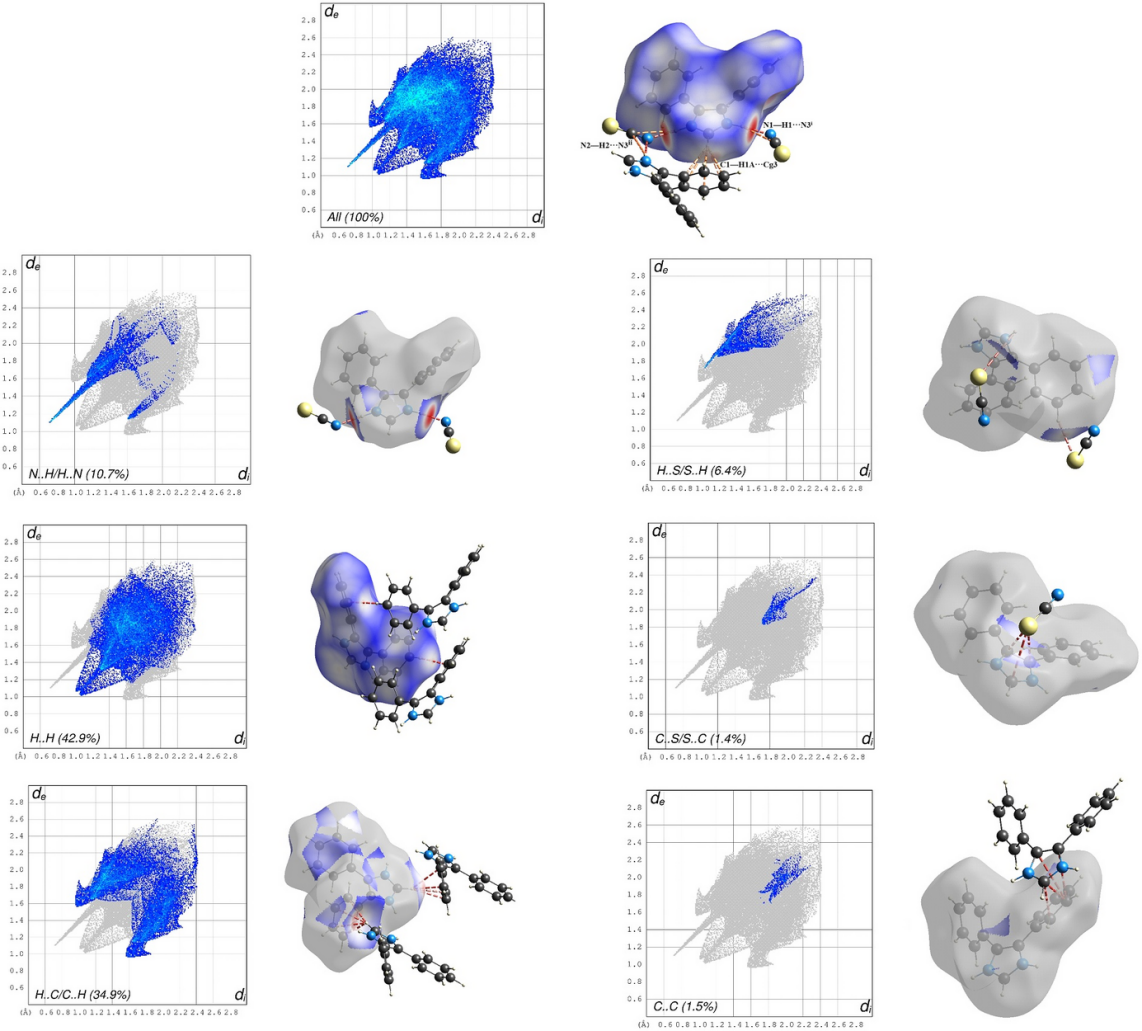
Hirshfeld surface mapped over *d*_norm_ and the associated fingerprint plots for (**I**), showing the percentage contributions of the different inter­molecular contacts.

Hirshfeld surface (HS) analysis and the corresponding two-dimensional fingerprint plots (Fig. 4 ▸) were generated using *CrystalExplorer 21.5* (Spackman *et al.*, 2021 ▸) in order to examine the inter­molecular inter­actions governing the crystal packing of the title salt. On the *d*_norm_-mapped Hirshfeld surface (Fig. 6), the most intense red spots are associated with the shortest inter­molecular contacts, particularly those involving the thio­cyanate anion. These include the N—H⋯N and C—H⋯S hydrogen bonds, as well as the C—S⋯π and C—H⋯π inter­actions, which play a central role in the cohesion of the ionic assembly.

The two-dimensional fingerprint plots indicate that H⋯H contacts make the largest contribution to the Hirshfeld surface, accounting for 42.9%, as expected from the high proportion of hydrogen atoms in the organic cation and the importance of van der Waals inter­actions in the crystal packing.

The H⋯C/C⋯H contacts are also significant, contributing 34.9%, and they correspond to C-H⋯π inter­actions involving the aromatic rings. The H⋯N/N⋯H and H⋯S/S⋯H contacts contribute 10.7% and 6.4%, respectively, reflecting the presence of classical and non-classical hydrogen bonds involving the thio­cyanate anion. In addition, the C⋯S/S⋯C contacts represent 1.4%, while the C⋯C contacts account for 1.5% of the surface.

The remaining inter­molecular contacts are only minor, confirming that the supra­molecular arrangement is governed primarily by hydrogen bonding, van der Waals inter­actions and aromatic contacts.

## 5. Database survey

A search of the Cambridge Structural Database (CSD, version 6.01, updated to February 2026; Groom *et al.*, 2016 ▸) for structures related to the title compound gave 1676 hits for organic, non-polymeric, error-free single-crystal entries containing the 4,5-di­aryl­imidazole framework. The closest neutral analogues are the polymorphs of 4,5-diphenyl-1*H*-imidazole [CSD refcodes OCUSUA (Stibrany *et al.*, 2001 ▸), OCUSUA01 (Stibrany *et al.*, 2004 ▸), OCUSUA02 (Batsanov *et al.*, 2004 ▸), OCUSUA03 (Kounavi *et al.*, 2012 ▸), OCUSUA04 (Rheingold, 2013 ▸)], together with related derivatives such as DPDMTH (King & Sengier, 1978 ▸), FASFAH01 (Fridman *et al.*, 2009 ▸), FASFAH02 (Huang, 2016 ▸) and FOVNIO (Zhang, 2009 ▸). More relevant to the present structure are the imidazolium salts GETDIT (Braddock *et al.*, 2006 ▸) and PASBEQ (Kaftory *et al.*, 1998 ▸), which demonstrate that protonated di­aryl­imidazolium species are known, although no thio­cyanate salt of a 4,5-bi­aryl­imidazolium cation corresponding to the title compound was identified.

## 6. Synthesis and crystallization

An equimolar aqueous solution of 4,5-bi­phenyl­imidazolium and potassium thio­cyanate was prepared in 10 ml of water at room temperature. The reaction mixture was stirred and heated at 343 K for 10 min, then left to evaporate slowly at room temperature. After a few weeks, colourless to white transparent single crystals of the title salt suitable for X-ray diffraction were obtained.

## 7. Refinement

Crystal data, data collection and structure refinement details are summarized in Table 2 ▸. Hydrogen atoms were placed in calculated positions and refined using a riding model, with N—H = 0.86 Å and C—H = 0.93 Å with *U*_iso_(H) = 1.2*U*_eq_(carrier) in all cases.

## Supplementary Material

Crystal structure: contains datablock(s) I, import. DOI: 10.1107/S2056989026005931/hb8228sup1.cif

Supporting information file. DOI: 10.1107/S2056989026005931/hb8228sup3.docx

Supporting information file. DOI: 10.1107/S2056989026005931/hb8228Isup3.cml

CCDC reference: 2559579

Additional supporting information:  crystallographic information; 3D view; checkCIF report

## Figures and Tables

**Table 1:** Hydrogen-bond geometry (Å, °) *Cg*1, *Cg*2 and *Cg*3 are the centroids of the N1/C1/N2/C2/C9, C3–C8 and C10–C15 rings, respectively.

*D*—H⋯*A*	*D*—H	H⋯*A*	*D*⋯*A*	*D*—H⋯*A*
N1—H1⋯N3^i^	0.86	1.98	2.835 (4)	171
N2—H2⋯N3^ii^	0.86	2.02	2.862 (3)	168
C12—H12⋯S1^iii^	0.93	2.93	3.785 (2)	155
C1—H1*A*⋯*Cg*3^iv^	0.93	2.45	3.330 (3)	158
C6—H6⋯*Cg*2^v^	0.93	2.93	3.581 (4)	128
C7—H7⋯*Cg*1^vi^	0.93	2.91	3.297 (4)	106

Symmetry codes: (i) 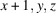; (ii) 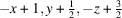; (iii) 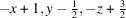; (iv) 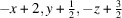; (v) 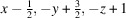; (vi) 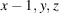.

**Table 2:** Experimental details

Crystal data	
Chemical formula	C_15_H_13_N_2_^+^·SCN^−^
*M*_r_	279.35
Crystal system, space group	Orthorhombic, *P*2_1_2_1_2_1_
Temperature (K)	100
*a*, *b*, *c* (Å)	6.5328 (3), 12.0855 (5), 18.0627 (8)
*V* (Å^3^)	1426.09 (11)
*Z*	4
Radiation type	Mo *K*α
μ (mm^−1^)	0.22
Crystal size (mm)	0.20 × 0.15 × 0.10

Data collection	
Diffractometer	Bruker APEXII CCD
Absorption correction	Multi-scan *CrysAlis PRO*; Agilent, 2014 ▸).
*T*_min_, *T*_max_	0.660, 1.000
No. of measured, independent and observed [*I* > 2σ(*I*)] reflections	8189, 2428, 2395
*R*_int_	0.018
(sin θ/λ)_max_ (Å^−1^)	0.595

Refinement	
*R*[*F*^2^ > 2σ(*F*^2^)], *wR*(*F*^2^), *S*	0.035, 0.084, 0.92
No. of reflections	2428
No. of parameters	169
No. of restraints	72
H-atom treatment	H-atom parameters constrained
Δρ_max_, Δρ_min_ (e Å^−3^)	0.23, −0.18
Absolute structure	Flack *x* determined using 919 quotients [(*I*^+^)−(*I*^−^)]/[(*I*^+^)+(*I*^−^)] (Parsons *et al.*, 2013 ▸)
Absolute structure parameter	0.08 (3)

Computer programs: *APEX3* and *SAINT* (Bruker, 2015 ▸), *SHELXT2018/2* (Sheldrick, 2015*a* ▸), *SHELXL2018/3* (Sheldrick, 2015*b* ▸) and *OLEX2* (Dolomanov *et al.*, 2009 ▸).

## supplementary crystallographic information

### 4,5-Diphenyl-1H-imidazol-3-ium thiocyanate Crystal data

**Table:**

C_15_H_13_N_2_^+^·SCN^−^	*D*_x_ = 1.301 Mg m^−^^3^
*M**_r_* = 279.35	Mo *K*α radiation, λ = 0.71073 Å
Orthorhombic, *P*2_1_2_1_2_1_	Cell parameters from 7394 reflections
*a* = 6.5328 (3) Å	θ = 2.2–27.0°
*b* = 12.0855 (5) Å	µ = 0.22 mm^−^^1^
*c* = 18.0627 (8) Å	*T* = 100 K
*V* = 1426.09 (11) Å^3^	Block, colourless
*Z* = 4	0.20 × 0.15 × 0.10 mm
*F*(000) = 584	

### 4,5-Diphenyl-1H-imidazol-3-ium thiocyanate Data collection

**Table:**

Bruker APEXII CCD diffractometer	2395 reflections with *I* > 2σ(*I*)
Radiation source: fine-focus sealed X-ray tube	*R*_int_ = 0.018
ω and phi scans	θ_max_ = 25.0°, θ_min_ = 2.3°
Absorption correction: multi-scan CrysAlis PRO; Agilent, 2014).	*h* = −7→7
*T*_min_ = 0.660, *T*_max_ = 1.000	*k* = −14→13
8189 measured reflections	*l* = −20→15
2428 independent reflections	

### 4,5-Diphenyl-1H-imidazol-3-ium thiocyanate Refinement

**Table:**

Refinement on *F*^2^	Hydrogen site location: inferred from neighbouring sites
Least-squares matrix: full	H-atom parameters constrained
*R*[*F*^2^ > 2σ(*F*^2^)] = 0.035	*w* = 1/[σ^2^(*F*_o_^2^) + (0.0369*P*)^2^ + 1.2893*P*] where *P* = (*F*_o_^2^ + 2*F*_c_^2^)/3
*wR*(*F*^2^) = 0.084	(Δ/σ)_max_ = 0.001
*S* = 0.92	Δρ_max_ = 0.23 e Å^−^^3^
2428 reflections	Δρ_min_ = −0.18 e Å^−^^3^
169 parameters	Absolute structure: Flack *x* determined using 919 quotients [(*I*^+^)-(*I*^-^)]/[(*I*^+^)+(*I*^-^)] (Parsons *et al.*, 2013)
72 restraints	Absolute structure parameter: 0.08 (3)

### 4,5-Diphenyl-1H-imidazol-3-ium thiocyanate Special details

**Table:**

Geometry. All esds (except the esd in the dihedral angle between two l.s. planes) are estimated using the full covariance matrix. The cell esds are taken into account individually in the estimation of esds in distances, angles and torsion angles; correlations between esds in cell parameters are only used when they are defined by crystal symmetry. An approximate (isotropic) treatment of cell esds is used for estimating esds involving l.s. planes.

### 4,5-Diphenyl-1H-imidazol-3-ium thiocyanate Fractional atomic coordinates and isotropic or equivalent isotropic displacement parameters (Å^2^)

**Table:**

	*x*	*y*	*z*	*U*_iso_*/*U*_eq_	
N2	0.8116 (4)	0.70567 (18)	0.70220 (12)	0.0231 (5)	
H2	0.756811	0.770267	0.704618	0.028*	
N1	1.0144 (4)	0.56874 (19)	0.72286 (13)	0.0234 (5)	
H1	1.113206	0.529875	0.740612	0.028*	
C2	0.7439 (4)	0.6191 (2)	0.65825 (14)	0.0199 (6)	
C3	0.5656 (4)	0.6301 (2)	0.60863 (15)	0.0215 (6)	
C9	0.8718 (4)	0.5323 (2)	0.67190 (15)	0.0209 (6)	
C1	0.9748 (5)	0.6726 (2)	0.73990 (15)	0.0249 (6)	
H1A	1.049153	0.715774	0.772989	0.030*	
C8	0.4006 (4)	0.6957 (2)	0.62873 (17)	0.0256 (6)	
H8	0.401379	0.732630	0.673906	0.031*	
C7	0.2342 (5)	0.7063 (3)	0.58137 (18)	0.0325 (7)	
H7	0.123673	0.750352	0.594930	0.039*	
C10	0.8736 (3)	0.41895 (12)	0.64286 (11)	0.0242 (6)	
C11.	0.6950 (3)	0.35642 (16)	0.64335 (12)	0.0364 (7)	
H11.	0.576452	0.384955	0.664493	0.044*	
C12	0.6937 (3)	0.25123 (16)	0.61222 (14)	0.0594 (10)	
H12	0.574200	0.209394	0.612546	0.071*	
C13	0.8709 (5)	0.20858 (14)	0.58061 (13)	0.0676 (11)	
H13	0.869996	0.138202	0.559791	0.081*	
C14	1.0495 (4)	0.27111 (18)	0.58013 (12)	0.0605 (10)	
H14	1.168045	0.242569	0.558982	0.073*	
C15	1.0508 (3)	0.37629 (16)	0.61125 (12)	0.0387 (7)	
H15	1.170301	0.418130	0.610928	0.046*	
C4	0.5655 (5)	0.5773 (2)	0.53980 (16)	0.0268 (7)	
H4	0.677352	0.534978	0.525256	0.032*	
C6	0.2324 (5)	0.6516 (3)	0.51417 (18)	0.0342 (8)	
H6	0.119540	0.657535	0.483036	0.041*	
C5	0.3983 (5)	0.5883 (2)	0.49340 (17)	0.0326 (8)	
H5	0.397810	0.552654	0.447776	0.039*	
S1	0.66610 (13)	0.50873 (8)	0.86212 (5)	0.0427 (3)	
N3	0.3105 (4)	0.4330 (2)	0.79293 (15)	0.0325 (6)	
C16	0.4590 (5)	0.4652 (2)	0.82209 (16)	0.0259 (7)	

### 4,5-Diphenyl-1H-imidazol-3-ium thiocyanate Atomic displacement parameters (Å^2^)

**Table:**

	*U*^11^	*U*^22^	*U*^33^	*U*^12^	*U*^13^	*U*^23^
N2	0.0267 (13)	0.0186 (11)	0.0241 (12)	0.0019 (10)	−0.0020 (11)	−0.0028 (10)
N1	0.0216 (12)	0.0244 (12)	0.0241 (12)	0.0022 (10)	−0.0042 (10)	0.0027 (10)
C2	0.0238 (14)	0.0168 (13)	0.0191 (13)	−0.0014 (11)	0.0009 (11)	0.0006 (10)
C3	0.0241 (14)	0.0153 (13)	0.0253 (15)	−0.0039 (12)	−0.0020 (11)	0.0042 (11)
C9	0.0215 (14)	0.0224 (14)	0.0188 (13)	−0.0028 (11)	−0.0008 (11)	0.0018 (11)
C1	0.0271 (15)	0.0259 (15)	0.0216 (15)	−0.0032 (13)	−0.0031 (12)	−0.0034 (11)
C8	0.0262 (15)	0.0225 (14)	0.0281 (16)	−0.0019 (12)	0.0033 (12)	0.0034 (12)
C7	0.0231 (15)	0.0324 (17)	0.0420 (18)	0.0015 (13)	−0.0003 (14)	0.0128 (14)
C10	0.0369 (14)	0.0180 (12)	0.0177 (13)	0.0048 (10)	−0.0048 (11)	0.0030 (11)
C11.	0.0415 (16)	0.0238 (13)	0.0439 (18)	−0.0009 (12)	−0.0213 (15)	0.0059 (13)
C12	0.093 (3)	0.0228 (15)	0.062 (2)	−0.0045 (16)	−0.053 (2)	0.0034 (14)
C13	0.142 (3)	0.0260 (17)	0.035 (2)	0.0237 (17)	−0.039 (2)	−0.0085 (15)
C14	0.116 (3)	0.0386 (17)	0.0265 (17)	0.0392 (17)	0.004 (2)	0.0023 (14)
C15	0.0556 (18)	0.0327 (14)	0.0278 (16)	0.0181 (14)	0.0119 (14)	0.0102 (12)
C4	0.0367 (17)	0.0175 (14)	0.0261 (15)	0.0018 (13)	−0.0032 (13)	0.0013 (11)
C6	0.0339 (17)	0.0316 (17)	0.0371 (17)	−0.0091 (14)	−0.0174 (15)	0.0155 (15)
C5	0.050 (2)	0.0197 (15)	0.0280 (16)	−0.0076 (14)	−0.0147 (15)	0.0024 (12)
S1	0.0329 (4)	0.0460 (5)	0.0491 (5)	−0.0121 (4)	−0.0116 (4)	0.0055 (4)
N3	0.0307 (14)	0.0248 (13)	0.0421 (15)	−0.0007 (12)	−0.0094 (13)	0.0036 (12)
C16	0.0318 (17)	0.0205 (14)	0.0254 (15)	0.0029 (13)	0.0010 (13)	0.0069 (11)

### 4,5-Diphenyl-1H-imidazol-3-ium thiocyanate Geometric parameters (Å, º)

**Table:**

N2—C1	1.326 (4)	C10—C15	1.3900
N2—C2	1.386 (3)	C11.—C12	1.3900
N2—H2	0.8600	C11.—H11.	0.9300
N1—C1	1.319 (4)	C12—C13	1.3900
N1—C9	1.382 (4)	C12—H12	0.9300
N1—H1	0.8600	C13—C14	1.3900
C2—C9	1.363 (4)	C13—H13	0.9300
C2—C3	1.476 (4)	C14—C15	1.3900
C3—C8	1.386 (4)	C14—H14	0.9300
C3—C4	1.397 (4)	C15—H15	0.9300
C9—C10	1.468 (3)	C4—C5	1.383 (4)
C1—H1A	0.9300	C4—H4	0.9300
C8—C7	1.389 (4)	C6—C5	1.379 (5)
C8—H8	0.9300	C6—H6	0.9300
C7—C6	1.382 (5)	C5—H5	0.9300
C7—H7	0.9300	S1—C16	1.622 (3)
C10—C11.	1.3900	N3—C16	1.171 (4)
			
C1—N2—C2	108.9 (2)	C15—C10—C9	119.98 (17)
C1—N2—H2	125.6	C10—C11.—C12	120.0
C2—N2—H2	125.6	C10—C11.—H11.	120.0
C1—N1—C9	109.0 (2)	C12—C11.—H11.	120.0
C1—N1—H1	125.5	C13—C12—C11.	120.0
C9—N1—H1	125.5	C13—C12—H12	120.0
C9—C2—N2	106.3 (2)	C11.—C12—H12	120.0
C9—C2—C3	131.6 (2)	C12—C13—C14	120.0
N2—C2—C3	122.1 (2)	C12—C13—H13	120.0
C8—C3—C4	119.6 (3)	C14—C13—H13	120.0
C8—C3—C2	120.4 (3)	C13—C14—C15	120.0
C4—C3—C2	120.0 (3)	C13—C14—H14	120.0
C2—C9—N1	106.8 (2)	C15—C14—H14	120.0
C2—C9—C10	131.2 (2)	C14—C15—C10	120.0
N1—C9—C10	122.0 (2)	C14—C15—H15	120.0
N1—C1—N2	108.9 (3)	C10—C15—H15	120.0
N1—C1—H1A	125.5	C5—C4—C3	119.7 (3)
N2—C1—H1A	125.5	C5—C4—H4	120.1
C3—C8—C7	120.0 (3)	C3—C4—H4	120.1
C3—C8—H8	120.0	C5—C6—C7	119.8 (3)
C7—C8—H8	120.0	C5—C6—H6	120.1
C6—C7—C8	120.2 (3)	C7—C6—H6	120.1
C6—C7—H7	119.9	C6—C5—C4	120.6 (3)
C8—C7—H7	119.9	C6—C5—H5	119.7
C11.—C10—C15	120.0	C4—C5—H5	119.7
C11.—C10—C9	119.93 (17)	N3—C16—S1	179.4 (3)
			
C1—N2—C2—C9	0.7 (3)	C2—C9—C10—C11.	−49.5 (4)
C1—N2—C2—C3	−179.2 (2)	N1—C9—C10—C11.	129.8 (2)
C9—C2—C3—C8	145.7 (3)	C2—C9—C10—C15	127.0 (3)
N2—C2—C3—C8	−34.4 (4)	N1—C9—C10—C15	−53.7 (3)
C9—C2—C3—C4	−36.5 (4)	C15—C10—C11.—C12	0.0
N2—C2—C3—C4	143.4 (3)	C9—C10—C11.—C12	176.5 (2)
N2—C2—C9—N1	−0.6 (3)	C10—C11.—C12—C13	0.0
C3—C2—C9—N1	179.2 (3)	C11.—C12—C13—C14	0.0
N2—C2—C9—C10	178.7 (3)	C12—C13—C14—C15	0.0
C3—C2—C9—C10	−1.4 (5)	C13—C14—C15—C10	0.0
C1—N1—C9—C2	0.4 (3)	C11.—C10—C15—C14	0.0
C1—N1—C9—C10	−179.1 (2)	C9—C10—C15—C14	−176.5 (2)
C9—N1—C1—N2	0.1 (3)	C8—C3—C4—C5	−2.0 (4)
C2—N2—C1—N1	−0.5 (3)	C2—C3—C4—C5	−179.7 (2)
C4—C3—C8—C7	1.7 (4)	C8—C7—C6—C5	−1.4 (5)
C2—C3—C8—C7	179.5 (3)	C7—C6—C5—C4	1.2 (5)
C3—C8—C7—C6	0.0 (4)	C3—C4—C5—C6	0.5 (4)

### 4,5-Diphenyl-1H-imidazol-3-ium thiocyanate Hydrogen-bond geometry (Å, º)

*Cg*1, *Cg*2 and *Cg*3 are the centroids of the N1/C1/N2/C2/C9, C3–C8 and C10–C15 rings, respectively.

**Table:**

*D*—H···*A*	*D*—H	H···*A*	*D*···*A*	*D*—H···*A*
N1—H1···N3^i^	0.86	1.98	2.835 (4)	171
N2—H2···N3^ii^	0.86	2.02	2.862 (3)	168
C12—H12···S1^iii^	0.93	2.93	3.785 (2)	155
C1—H1*A*···*Cg*3^iv^	0.93	2.45	3.330 (3)	158
C6—H6···*Cg*2^v^	0.93	2.93	3.581 (4)	128
C7—H7···*Cg*1^vi^	0.93	2.91	3.297 (4)	106

Symmetry codes: (i) *x*+1, *y*, *z*; (ii) −*x*+1, *y*+1/2, −*z*+3/2; (iii) −*x*+1, *y*−1/2, −*z*+3/2; (iv) −*x*+2, *y*+1/2, −*z*+3/2; (v) *x*−1/2, −*y*+3/2, −*z*+1; (vi) *x*−1, *y*, *z*.
