# Supplementary material for: Synthesis and structure of 4,5-diphenyl-1H-imidazol-3-ium thio­cyanate
Source: Acta Crystallogr E Crystallogr Commun. 2026 Jun 9;82(Pt 7):811–5. doi: 10.1107/S2056989026005931 (PMC13330904; doi:10.1107/S2056989026005931)
Supplement: Supplementary file 2 [file e-82-00811-sup3.docx]

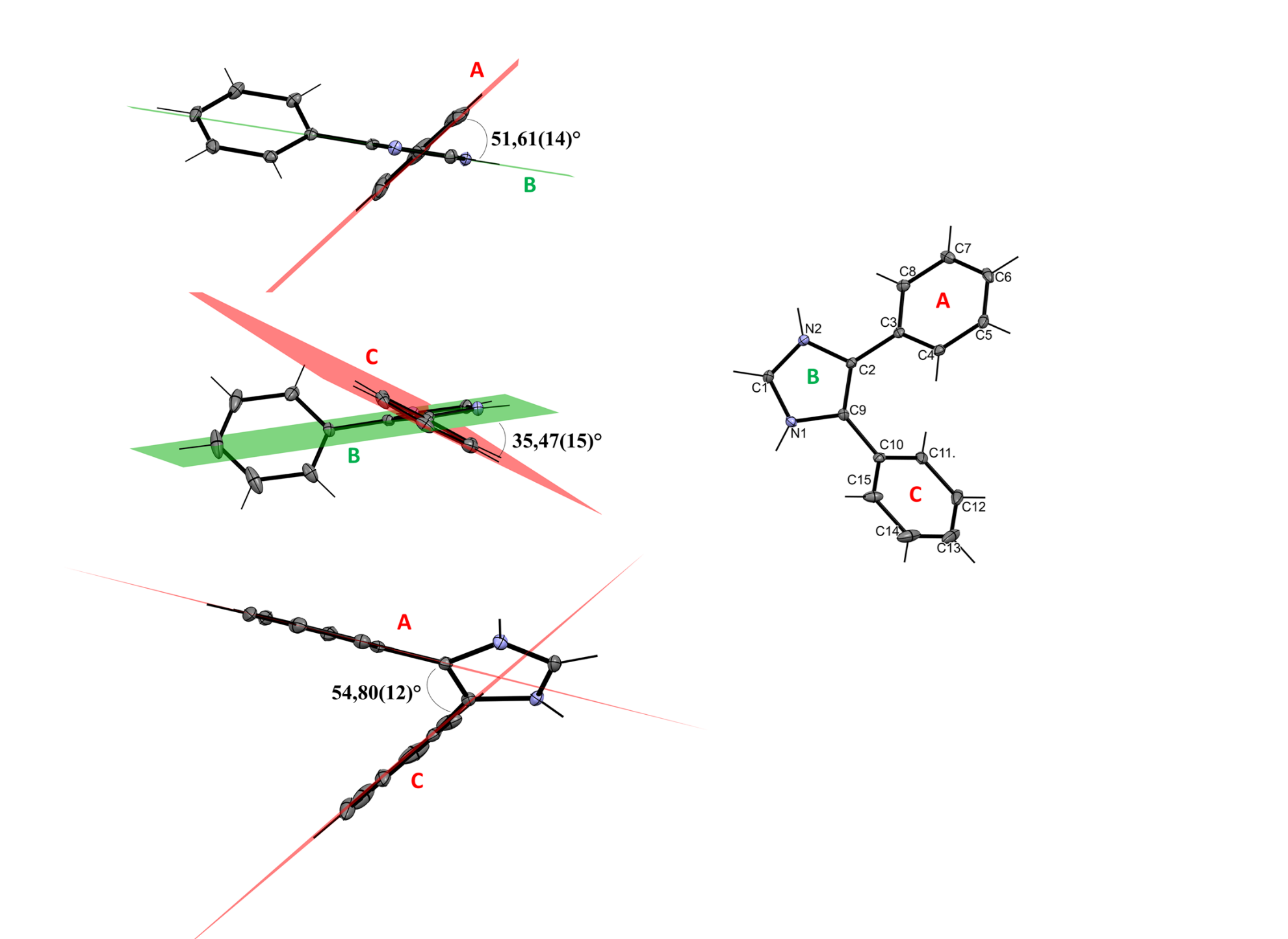


**Figure S1.** Molecular geometry of the cation showing the relative orientations of the three ring systems labelled A, B and C; the interplanar angles are A/B = 51.61 (14)°, B/C = 35.47 (15)° and A/C = 54.80 (12)°.


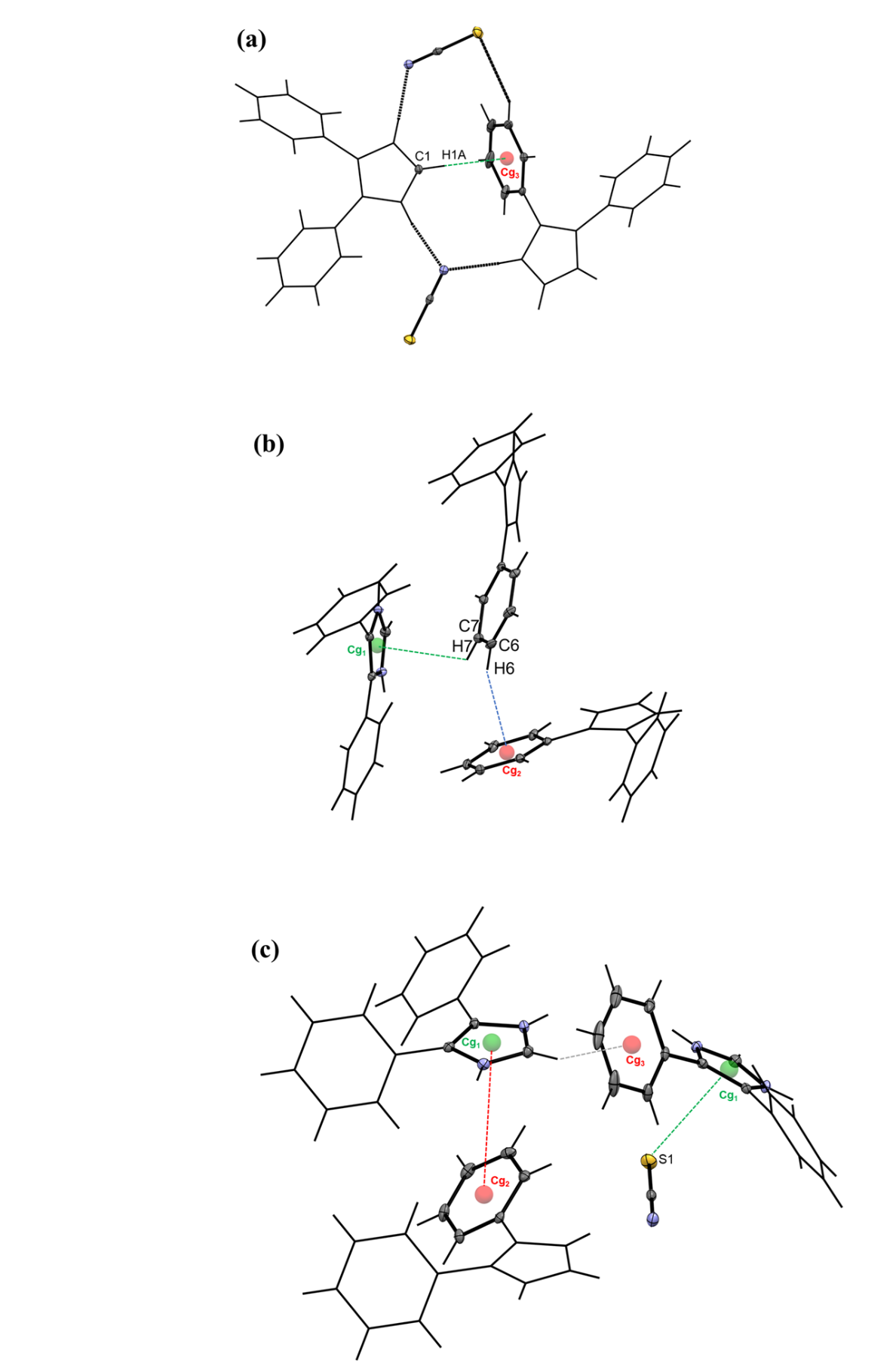


**Figure S1.** Supramolecular features in the crystal packing: **(a)** C-H···π interaction C1-H1A···Cg3 reinforcing the $R_{4}^{3}(16)$ motif and the associated chain-forming contacts; **(b)** additional C—H···π contacts involving Cg1 and Cg2; and **(c)** weak π–π stacking between the A and B aromatic rings, with a centroid–centroid separation of 4.201(12) Å, reinforced by a C—S···Cg1 contact [3.534(2) Å, 94.96(11)°].
